# Global, regional, and national burden of ovarian cancer from 1990 to 2021: Analysis of risk factors and prediction of trends in 2050

**DOI:** 10.1097/MD.0000000000050828

**Published:** 2026-09-25

**Authors:** Chaokang Huang, Xin Zheng, Yajing Zhang, Xike Zhang, Jian Zhang

**Affiliations:** a Department of Pathology, Xingtai Peoples Hospital, Hebei, China; b Department of Endocrinology, Xingtai Peoples Hospital, Hebei, China.

**Keywords:** ARIMA model, cancer disparities, Global Burden of Disease, gynecological oncology, ovarian cancer, public health, socio-demographic Index

## Abstract

Ovarian cancer is one of the most lethal gynecological malignancies globally, with significant variations in incidence, prevalence, and outcomes across regions and population subgroups. This study systematically evaluates the global, regional, and national burden of ovarian cancer from 1990 to 2021, utilizing data from the Global Burden of Disease 2021 framework. Trends in incidence, prevalence, mortality, and disability-adjusted life years were analyzed, with an emphasis on their associations with the Socio-Demographic Index (SDI) and other key risk factors. The analysis highlights substantial disparities in ovarian cancer burden, with low- and middle-SDI regions experiencing the highest mortality-to-incidence ratios. In 2021, ovarian cancer accounted for approximately 298,876 new cases globally, with age-standardized incidence rates declining in high-SDI regions but increasing in low-SDI regions. Mortality trends mirrored this pattern, with significant disparities between high- and low-resource settings. The findings underscore the critical influence of age, genetics, and access to early detection and treatment. Forecasts using an ARIMA model indicate a continued rise in the global burden, particularly in regions with limited healthcare resources. This study underscores the urgent need for targeted interventions to improve ovarian cancer outcomes, including enhanced screening, equitable access to treatment, and strategies addressing modifiable risk factors, particularly in underserved regions.

## 1. Introduction

Ovarian cancer is a malignant condition originating in the ovarian epithelium or surrounding tissues and represents a leading cause of cancer-related mortality in women worldwide. Despite accounting for only 3% of all cancers in women, ovarian cancer has a disproportionately high mortality-to-incidence ratio due to its typically asymptomatic progression and late-stage diagnosis.^[1]^ Globally, the disease poses a significant burden, with over 298,876 new cases and 185,608 deaths reported in 2021 alone. These statistics highlight an urgent need to improve early detection, optimize treatment approaches, and reduce disparities in outcomes.

The risk of developing ovarian cancer is influenced by a combination of genetic, environmental, and lifestyle factors, with BRCA1 and BRCA2 mutations representing major contributors to hereditary risk.^[2,3]^ Other risk factors include advanced age, nulliparity, endometriosis, and hormonal imbalances, while protective factors such as oral contraceptive use and parity may lower risk.^[4]^ Despite advances in surgical techniques and targeted therapies, 5-year survival rates remain below 50% in many countries, reflecting challenges in early detection and treatment accessibility.^[4,5]^

Disparities in ovarian cancer incidence and outcomes are closely tied to socioeconomic factors, healthcare infrastructure, and public health priorities. High- socio-demographic index (SDI)regions have seen declining incidence rates, likely due to preventive measures and improved risk assessment, whereas low-SDI regions continue to experience rising burdens, exacerbated by limited healthcare access and inadequate awareness.^[5,6]^ Furthermore, the aging global population is projected to contribute to a significant increase in the disease burden over the coming decades.^[7]^

This study aims to systematically assess the global burden of ovarian cancer, examining trends in incidence, prevalence, mortality, and disability-adjusted life year (DALYs) using the Global Burden of Disease (GBD) framework from 1990 to 2021. By analyzing these trends across SDI levels, geographic regions, and age groups, this study seeks to identify actionable insights for policymakers to allocate resources effectively and implement equitable strategies to combat ovarian cancer. Additionally, prospective forecasting using an ARIMA model provides critical insights into future trends, informing targeted interventions to reduce disparities and improve outcomes globally.

## 2. Materials and methods

This study systematically evaluated the global burden of ovarian cancer, including its incidence, prevalence, DALYs, and mortality, from 1990 to 2021, using the GBD 2021 framework. The research methods are detailed as follows:

### 2.1. Data sources

Data for this analysis were derived from the GBD 2021 database on the Global Health Data Exchange (GHDx) platform.^[8]^ The database includes comprehensive epidemiological data on 369 diseases and injuries across 204 countries and territories. Metrics analyzed include incidence, prevalence, DALYs, and mortality, as well as their age-standardized rates.

### 2.2. Case definition

Ovarian cancer was defined as malignant neoplasms of the ovary; ICD-10 code C56, 10th Revision (ICD-10, according to the International Classification of Diseases).^[9]^ Data sources incorporated registries, hospital records, and population-based studies, harmonized through the GBD framework to ensure consistency and comparability across countries and time periods.

### 2.3. Data processing and statistical analysis

R software (v4.3.3) was used for data analysis. Mixed-effects modeling was employed to analyze variations in ovarian cancer burden across different SDI regions. The SDI categorizes regions into 5 levels based on per capita income, educational attainment, and fertility rates: low SDI (<0.46), low-middle SDI (0.46–0.60), middle SDI (0.61–0.69), high-middle SDI (0.70–0.81), and high SDI (>0.81).

To account for heterogeneity in data sources, Bayesian meta-regression with the DisMod-MR 2.1 tool was used to integrate diverse datasets and estimate disease-specific parameters. Uncertainty intervals (UI) for metrics were calculated from the median values of 1000 Monte Carlo simulations between the 25th and 975th values, providing robust estimates for each indicator.

The Age-Standardized Rate was used to evaluate the burden and differences in periodontal disease between countries and regions. Estimated Annual Percentage Change (EAPC) was used to calculate the average annual change rate for age-standardized prevalence, incidence, and DALYs to assess the temporal trends in periodontal disease. The formula for the model is:


$$\begin{array}{c} EAPC=\left(e^{\beta }-1\right)\times 100\%, \end{array}$$ (1)

where *β* is the slope of the regression line, which represents the change in the log of the age-standardized burdens over time.

All statistical tests were 2-sided, and a significance level of *α* = 0.05 was applied.

### 2.4. Forecasting future burden

To predict future trends in ovarian cancer burden, an Autoregressive Integrated Moving Average (ARIMA) model was applied using data from 1990 to 2021. The model accounted for historical patterns and projected incidence, prevalence, DALYs, and mortality through 2050, with an emphasis on regional and SDI disparities.

### 2.5. Ethical considerations

This study utilized publicly available, de-identified data, and as such, ethical approval was not required. The study adhered to the “Guidelines for Accurate and Transparent Health Estimates Reporting” (GATHER) to ensure methodological transparency and reliability.

## 3. Results

### 3.1. Global and regional burden of ovarian cancer (1990–2021)

From 1990 to 2021, the global profile of ovarian cancer changed unevenly across countries and socioeconomic strata. In 2021, approximately 298,876 new cases of ovarian cancer were recorded worldwide (95% UI: 270,730–324,501), corresponding to an age-standardized incidence rate (ASIR) of 6.71 per 100,000 (95% UI: 6.07–7.28). Although the number of cases has grown steadily, the standardized rate showed a mild overall decline over the past 3 decades (EAPC = –0.38%, 95% UI:–0.43 to–0.32). A similar downward pattern was observed for mortality. About 185,609 deaths (95% UI: 167,962–201,013) occurred in 2021, giving an age-standardized mortality rate (ASMR) of 4.06 per 100,000 (95% UI: 3.67–4.40) and an EAPC of–0.40% (95% UI:–0.47 to–0.32) (Table 1).

**Table 1:** Global ovarian cancer incidence, prevalence, disability-adjusted life years (DALYs), and mortality rates by gender in 2021, including age-standardized rates and estimated annual percentage changes (EAPC).

	2021		2021		2021		2021		EAPC 1990-2021			
	Incidence	ASR (per 100,000)	Prevalence	ASR (per 100,000)	DALYs	ASR (per 100,000)	Deaths	ASR (per 100,000)	ASIR	ASPR	ASDR	ASMR
Global	298,876 (270,729.82– 324,501.02)	6.71 (6.07 –7.28)	1,222,425.26 (1,102,105.85 –1,332,111.98)	28.08 (25.26 –30.64)	5,163,256.3 (4,692,422.55– 5,608,304.11)	115.15 (104.58 –125.21)	185,608.68 (167,961.98 –201,012.67)	4.06 (3.67 –4.4)	−0.38% (−0.43%– −0.32%)	−0.07% (−0.13% −0.00%)	−0.59% (−0.64% – −0.54%)	−0.40% (−0.47% – −0.32%)
Low SDI	17,466.29 (13,144.54 –20,886.84)	5.42 (4.12 – 6.43)	69,016.22 (51,214.74 – 83,333.42)	18.42 (13.85 – 22.07)	345,369.48 (259,740.87 –413,461.42)	108.72 (82.67 – 129.4)	10,381.91 (7906.56 – 12,360.53)	3.74 (2.87 –4.43)	1.18% (1.09% – 1.28%)	1.53% (1.42% –1.64%)	0.99% (0.89% –1.08%)	0.98% (0.92% –1.03%)
Low-middle SDI	50,273.66 (43,482.3 –59,052.33)	6.01 (5.22 – 7.07)	204,118.93 (175,156.39 – 239,874.19)	22.92 (19.71– 26.94)	925,850.81 (802,377.33– 1,091,390.66)	111.4 (96.54 – 131.2)	28,936.78 (25,193.38 –34,128.96)	3.72 (3.24 –4.4)	1.51% (1.45% –1.58%)	1.88% (1.81% –1.94%)	1.29% (1.23% –1.34%)	1.44% (1.40% –1.49%)
Middle SDI	82,367.53 (71,878.65 –92,302.66)	5.81 (5.07 – 6.5)	356,646.19 (306,136.48 –400,487.44)	25.43 (21.73 – 28.62)	1,416,148.86 (1,244,404.1– 1,587,044.09)	97.76 (85.77 –109.48)	45,543.21 (39,960.19 –51,216.78)	3.18 (2.78 –3.57)	0.73% (0.65% –0.81%)	1.24% (1.16% –1.31%)	0.31% (0.22% – 0.40%)	0.56% (0.48% –0.65%)
High-middle SDI	67,777.96 (60,033.34 – 75,752.67)	6.93 (6.1 –7.76)	275,500.06 (241,626.12 –309,285.56)	30.37 (26.64 –34.23)	1,194,242.16 (1,054,540 –1,335,946.43)	119.79 (105.13 –134.14)	44,090.83 (38,883.74 –48,926.94)	4.16 (3.68 –4.63)	−0.53% (−0.62%– −0.45%)	−0.22% (−0.29% –−0.15%)	−0.87% (−0.96% –−0.79%)	−0.32% (−0.45% – −0.19%)
High SDI	80,651.37 (73,424.59 –85,364.17)	8.4 (7.84 – 8.81)	315,838.23 (296,353.55 –330,296.1)	37.89 (35.88 –39.41)	1,275,650.43 (1,173,209.42– 1,343,084.89)	133.8 (125.32 –140.06)	56,426.76 (50,328.49 –60,245.8)	5.08 (4.62 –5.37)	−1.21% (−1.33% –−1.09%)	-0.94% (−1.10% –−0.78%)	-1.48% (−1.57% –−1.39%)	-1.04% (−1.14%–−0.93%)
Central Asia	2998.96 (2602.07– 3418.89)	6.1 (5.31– 6.94)	12,119.22 (10,475.23 –13,837.45)	24.01 (20.78 – 27.37)	61,881.11 (53,807.39 –70,556.41)	124.11 (108.08 –141.31)	1949.03 (1705.34 – 2219.78)	4.08 (3.57 –4.63)	1.22% (1.06% –1.38%)	1.30% (1.14% –1.47%)	1.05% (0.90% –1.21%)	1.32% (1.22% –1.42%)
East Asia	44,239.91 (33,123.68 –57,358.78)	4.21 (3.15 –5.46)	193,221.83 (143,594.41 –251,411.89)	19.58 (14.56 ––25.49)	787,095.89 (580,908.59– 1,024,657.54)	72.07 (53.13 –94.15)	26,337.19 (19,516.44 –34,082.62)	2.33 (1.72 –3.01)	-0.35% (−0.49% –−0.20%)	0.37% (0.25% –−0.48%)	-0.92% (−1.09% –−0.74%)	-0.43% (−0.61% –−0.26%)
High-income Asia Pacific	11,227.65 (9782.4 –12,235.43)	6.36 (5.69 –6.75)	49,705.98 (44,069.34 –53,166.75)	33.22 (30.01 –35.16)	168,116.95 (148,704.1 –180,427.51)	98.97 (89.26 –104.57)	7200.86 (6064.3– 7968.63)	3.26 (2.87– 3.5)	−0.01% (−0.16% – 0.15%)	0.55% (0.31% –0.79%)	-0.63% (−0.70% –−0.55%)	-0.14% (−0.26% – −0.01%)
South Asia	49,664.47 (42,260.25 –59,355)	5.99 (5.13 –7.14)	193,424.48 (160,661.77 – 230,736.76)	22.03 (18.49 –26.24)	960,207.82 (822,850.63–1,142,385.68)	116 (99.82 –138.16)	30,585.13 (26,424.78– 36,402.65)	3.92 (3.39 – 4.67)	1.37% (1.27% –1.46%)	1.77% (1.68% −1.86%)	1.09% (1.00% −1.18%)	1.29% (1.22% −1.35%)
Southeast Asia	35,031.98 (26,271.21 –45,639.37)	9.28 (6.97 – 12.04)	166,779.53 (122,984.96 –216,888.66)	43.52 (32.08 –56.58)	484,774.21 (368,984.4 – 633,791.17)	126.63 (96.38 –165.18)	14,523.22 (11,286.29 –18,865.47)	3.96 (3.07 –5.12)	1.39% (1.27% –1.50%)	1.83% (1.69% –1.97%)	0.99% (0.91% –1.07%)	1.30% (1.21% –1.40%)
Australasia	1825.52 (1613 –1993.81)	7.18 (6.51– 7.81)	7225.03 (6585.66 –7833.95)	32.16 (29.64 –34.6)	29,401.76 (26,399.3– 31,858.63)	116.72 (106.15 –125.99)	1336.81 (1147.13 –1464.84)	4.68 (4.12 –5.08)	−2.21% (−2.58% – −1.84%)	−2.04% (−2.46% – −1.62%)	−2.50% (−2.83% –−2.16%)	−1.46% (−1.77% –−1.15%)
Oceania	185.52 (105.31– 256.55)	3.9 (2.32 – 5.28)	839.43 (462.36 –1185.97)	15.47 (8.78– 21.5)	2755.14 (1580.22 –3789.35)	59.04 (35.09 –80.86)	78.06 (46.55– 106.68)	1.97 (1.23– 2.64)	1.14% (1.01% –1.26%)	1.38% (1.23% –1.53%)	1.00% (0.90% –1.09%)	1.17% (1.09% –1.24%)
Central Europe	11,442.7 (10,515.96 –12,322.48)	10.8 (9.93 – 11.68)	39,849.81 (36,674.68 –43,037.77)	43.47 (39.98 –47.08)	215,729.52 (198,220.72 –232,085.81)	205.22 (189.37– 221.33)	8859.82 (8105.86 – 9520.21)	7.4 (6.79 – 7.96)	0.00% (−0.17% to 0.17%)	0.09% (−0.11% to 0.29%)	−0.20% (−0.36% –−0.04%)	0.32% (0.21% –0.43%)
Eastern Europe	18,015.49 (16,129.06 –20,269.46)	9.83 (8.77 to 11.03)	67,894.23 (60,235.42 –76,729.59)	41.71 (36.9 –47.34)	332,151.87 (296,367.31 –371,968.36)	180.89 (160.79– 203.55)	12,184.22 (10,926.45 –13,636.73)	6 (5.37– 6.73)	−0.22% (−0.40% –−0.04%)	−0.14% (−0.31% –−0.03%)	−0.44% (−0.62%– −0.25%)	0.23% (0.06% –0.40%)
Western Europe	38,281.57 (34,487.65 –40,724.83)	8.99 (8.35 –9.44)	145,908.44 (136,222.69 –153,009.27)	40.81 (38.95 –42.49)	594,073.8 (542,482.42 –629,975.05)	140.16 (131.08 –146.93)	28,235.78 (24,788.44 –30,470.63)	5.55 (5.03 –5.9)	−1.35% (−1.44%– −1.25%)	−1.13% (−1.27%– 0.98%)	−1.61% (−1.68% –−1.54%)	−1.06% (−1.16% –−0.96%)
Andean Latin America	2233.17 (1706.37 –2815.95)	6.9 (5.27 –8.68)	9484.99 (7149.38 –12,107.31)	28.51 (21.49 – 36.37)	40,484.05 (30,833.26 –50,874.95)	125.6 (95.71 –158.01)	1327.23 (1025.2 –1651.42)	4.22 (3.27 –5.26)	2.12% (1.72% –2.52%)	2.74% (2.29% –3.19%)	1.80% (1.43% –2.17%)	1.84% (1.63% –2.06%)
Central Latin America	11,429.14 (9936.69 – 12,916.31)	8.23 (7.16 –9.29)	48,879.47 (42,055.38 –55,319.41)	34.93 (30.06 –39.55)	210,430.41 (183,006.47 –237,783.39)	150.76 (131.25– 170.19)	6732.08 (5926.98 –7564.8)	4.9 (4.32 –5.5)	1.38% (1.29% –1.47%)	1.89% (1.78% –1.99%)	1.19% (1.11% –1.26%)	0.77% (0.67% –0.87%)
High-income North America	27,976.06 (25,614.91 –29,326.34)	8.88 (8.29 –9.27)	107,116.36 (101,112.07 –111,536.32)	38.38 (36.76 – 39.93)	439,443.86 (409,029.05 –459,505.05)	139.13 (131.42 –145.12)	19,233.43 (17,244.73 –20,306.07)	5.44 (4.96 –5.71)	−1.33% (−1.51% –-1.15%)	−1.30% (−1.49% –−1.11%)	−1.49% (−1.65% –−1.33%)	−0.92% (−1.07% –−0.77%)
Southern Latin America	3405.19 (3154.7 –3641.43)	7.69 (7.14 –8.23)	13,171.68 (12,192.5 –14,079.2)	31.73 (29.3 –33.94)	60,642.08 (56,166.79 –64,794)	137.3 (127.41 –146.65)	2284.02 (2093.7 –2454.77)	4.82 (4.44 –5.17)	−0.25% (−0.43% –−0.08%)	0.18% (−0.02%– −0.38%)	−0.51% (−0.67% –−0.36%)	−0.45% (−0.55% – −0.35%)
Tropical Latin America	8891.08 (8351.9 –9346.7)	6.43 (6.05 –6.77)	35,766.11 (33,789.08 –37,495.84)	26.41 (24.97 –27.71)	163,847.06 (153,851.82 –172,299.29)	117.39 (110.28 –123.42)	5608.42 (5170.52 –5934.15)	3.97 (3.67 –4.2)	0.20% (0.09%– 0.32%)	0.59% (0.48% –0.69%)	−0.02% (−0.13% –0.08%)	0.26% (0.16% –0.36%)
Caribbean	1830.92 (1569.51 –2215.28)	6.66 (5.7 –8.07)	7184.29 (6120.09 –8675.07)	26.86 (22.83 –32.64)	34,326.12 (29,349.77 –41,699.87)	124.35 (106 –151.66)	1182.62 (1018.56 –1400.18)	4.17 (3.59 –4.95)	0.91% (0.73% –1.09%)	1.17% (0.94% –1.39%)	0.80% (0.65% –0.96%)	0.95% (0.85% –1.06%)
North Africa and Middle East	12,436.21 (10,098.97 –14,422.59)	4.87 (3.96 –5.63)	54,349.68 (43,232.71 –62,929.83)	19.5 (15.67 –22.61)	225,627.85 (183,817.83 –261,748.07)	88.12 (71.92 –102.25)	7245.72 (5922.15 –8441.18)	3.14 (2.54 –3.65)	1.26% (1.19% –1.32%)	1.63% (1.55% –1.70%)	0.95% (0.88% –1.01%)	1.23% (1.17% –1.29%)
Central subSaharan Africa	1636.84 (949.44 –2332.89)	4.38 (2.49 –6.25)	6348.86 (3707.89 –8953.58)	14.6 (8.47 –20.94)	33,664.58 (19,263.61 –49,080.06)	90.01 (50.59 –129.58)	998.05 (560.9 – 1433.24)	3.1 (1.69 –4.42)	1.49% (1.28% –1.70%)	1.84% (1.58% –2.10%)	1.37% (1.17% –1.57%)	1.05% (0.91% –1.19%)
Eastern subSaharan Africa	8615.51 (5976.33 – 10,747.85)	7.48 (5.31 – 9.25)	34,898.2 (23,582.79 –44,024.45)	25.41 (17.53 –31.71)	170,255.6 (117,552.17 –211,891.34)	150.96 (106.18 –185.66)	5019.73 (3547.73 −6178.83)	5.21 (3.74 –6.35)	1.08% (0.99% –1.17%)	1.49% (1.37% –1.60%)	0.91% (0.83% –0.99%)	0.81% (0.75% –0.87%)
Southern subSaharan Africa	2775.58 (2119.3 –3220.22)	7.71 (5.88 –8.93)	10,011.12 (7740.63 –11,769.84)	26.13 (20.12 –30.57)	54,378.62 (41,720.33 –63,224.31)	150.23 (114.98 –174.11)	1819.39 (1377.45 –2091.06)	5.38 (4.05– 6.16)	1.61% (1.47% –1.74%)	1.55% (1.44% –1.67%)	1.59% (1.45% –1.73%)	1.50% (1.42% –1.59%)
Western Sub-Saharan Africa	4732.53 (3184.53 –6134.02)	3.62 (2.4 –4.64)	18,246.53 (12,264.98 –23,711.97)	11.78 (7.92 –15.22)	93,968.02 (63,290.63 –121,185.07)	72.64 (48.64 –93.39)	2867.87 (1906.15 –3686.2)	2.58 (1.69– 3.28)	1.59% (1.50% –1.68%)	1.84% (1.72% –1.95%)	1.46% (1.37% –1.55%)	1.39% (1.34% –1.44%)

Values in parentheses represent the 95% uncertainty intervals (UI).ASR, age-standardized rate (per 100,000 population); ASDR, age-standardized DALY rate; ASIR, age-standardized incidence rate; ASMR, age-standardized mortality rate; ASPR, age-standardized prevalence rate; EAPC, estimated annual percentage change.

Despite these modest declines, the overall disease burden remains substantial, with 5.16 million DALYs (95% UI: 4.69–5.61 million) and an age-standardized DALY rate (ASDR) of 115.15 per 100,000 (95% UI: 104.58–125.21). Global prevalence was estimated at 1.22 million cases (95% UI: 1.10–1.33 million), yielding an age-standardized prevalence rate of 28.08 per 100,000 (95% UI: 25.26–30.64).

Marked differences emerged when data were examined by Socio-Demographic Index (SDI). High-SDI regions reported the highest burden in 2021 (ASIR = 8.40; ASMR = 5.08; ASDR = 133.8 per 100,000), but these areas also demonstrated the sharpest reductions since 1990, with ASIR and ASMR decreasing by–1.21% and–1.04% per year, respectively. In contrast, low-middle-SDI regions showed the fastest growth in all indicators, with EAPCs of +1.51% for incidence and +1.44% for mortality. Middle-SDI regions had the lowest DALY and death rates, while low-SDI regions displayed the lowest incidence and prevalence.

At the country level, the gradient was even clearer. Palau, Kiribati, and Mali showed the smallest age-standardized rates for incidence (as low as 1.5 per 100,000) and prevalence (5.5–6.3 per 100,000), whereas the United Arab Emirates exhibited the highest figures, with incidence reaching 25.4 per 100,000. Island nations such as Seychelles and Mauritius also reported unusually high prevalence, exceeding 80 per 100,000. Overall, these findings illustrate an improving picture in high-income regions but a worrisome rise in burden where healthcare access remains limited.

### 3.2. Age-related distribution of ovarian cancer burden

The global age distribution in 2021 showed a gradual rise in ovarian cancer burden with advancing age. Incidence began to increase from ages 15 to 19 and accelerated notably after age 40, peaking around 90 to 94 years, then slightly declining beyond 95 years. Prevalence increased more steadily and reached its maximum at 60 to 64 years, after which it declined progressively. DALYs followed a similar pattern, increasing rapidly from early adulthood and reaching their highest level at 70 to 74 years. Mortality also rose with age, showing a slow increase up to age 39 and a marked rise thereafter, stabilizing in women older than 75. These results indicate that the disease mainly affects older women, though its burden is becoming increasingly apparent among middle-aged populations in less developed areas (Fig. 1).

**Figure 1.**
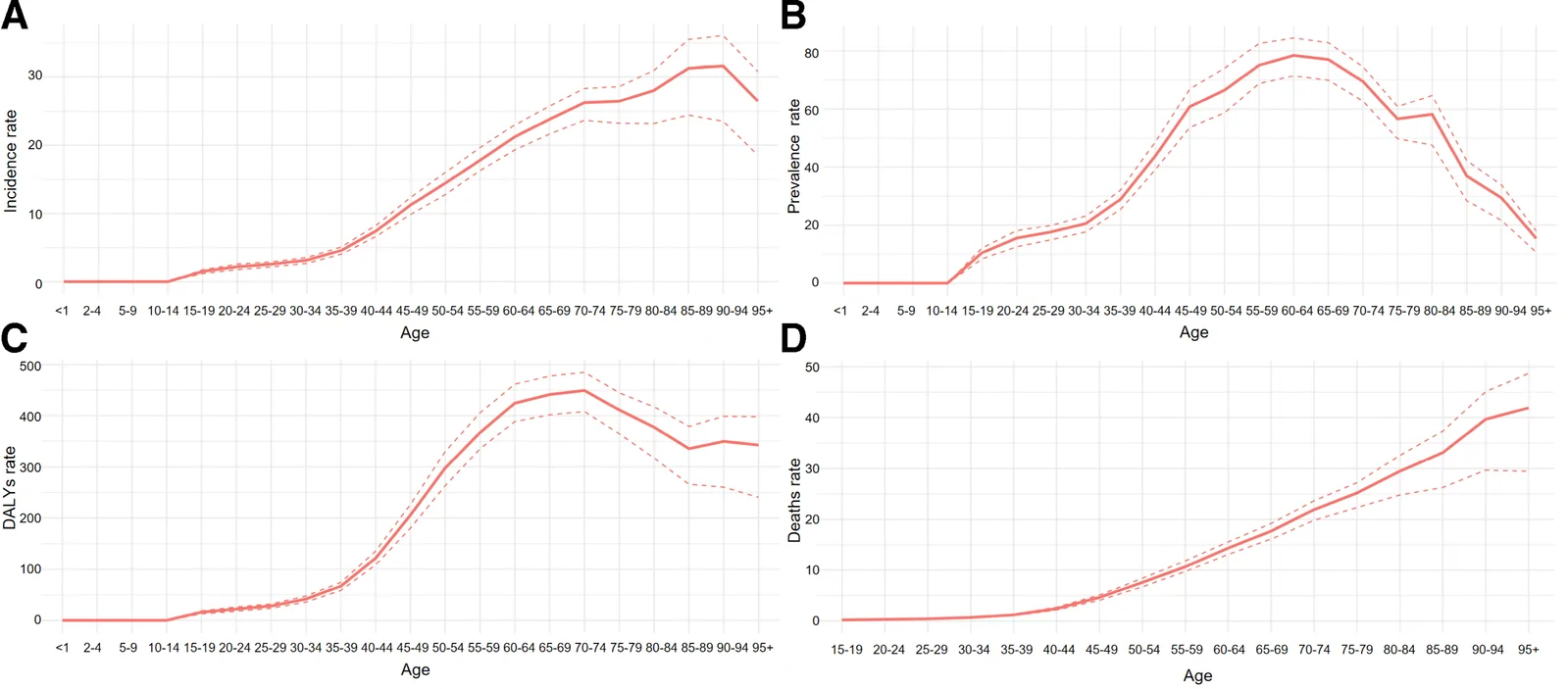
Global burden of ovarian cancer across different age groups in 2021 (dashed lines indicate 95% UI). (A) Incidence rate, (B) Prevalence rate, (C) DALY rate, (D) Mortality rate (per 100,000 population).DALY = disability-adjusted life year.

### 3.3. Relationship between ovarian cancer burden and SDI

As shown in Figure 2, the association between ovarian cancer burden and SDI was nonlinear. Regions with SDI values between 0.71 and 0.82 typically transitioning economies carried the heaviest burden, with the highest age-standardized rates of incidence, prevalence, DALYs, and mortality. Conversely, areas with SDI values below 0.45, such as Western subSaharan Africa, displayed the lowest levels. Australasia stood out with persistently high rates among developed regions, while Oceania had relatively low mortality and DALY rates. From 1990 to 2021, regions with SDI < 0.6 showed a steady rise in all indicators, whereas those with SDI > 0.7 demonstrated continuous declines, reflecting both healthcare improvements and demographic transitions.

**Figure 2.**
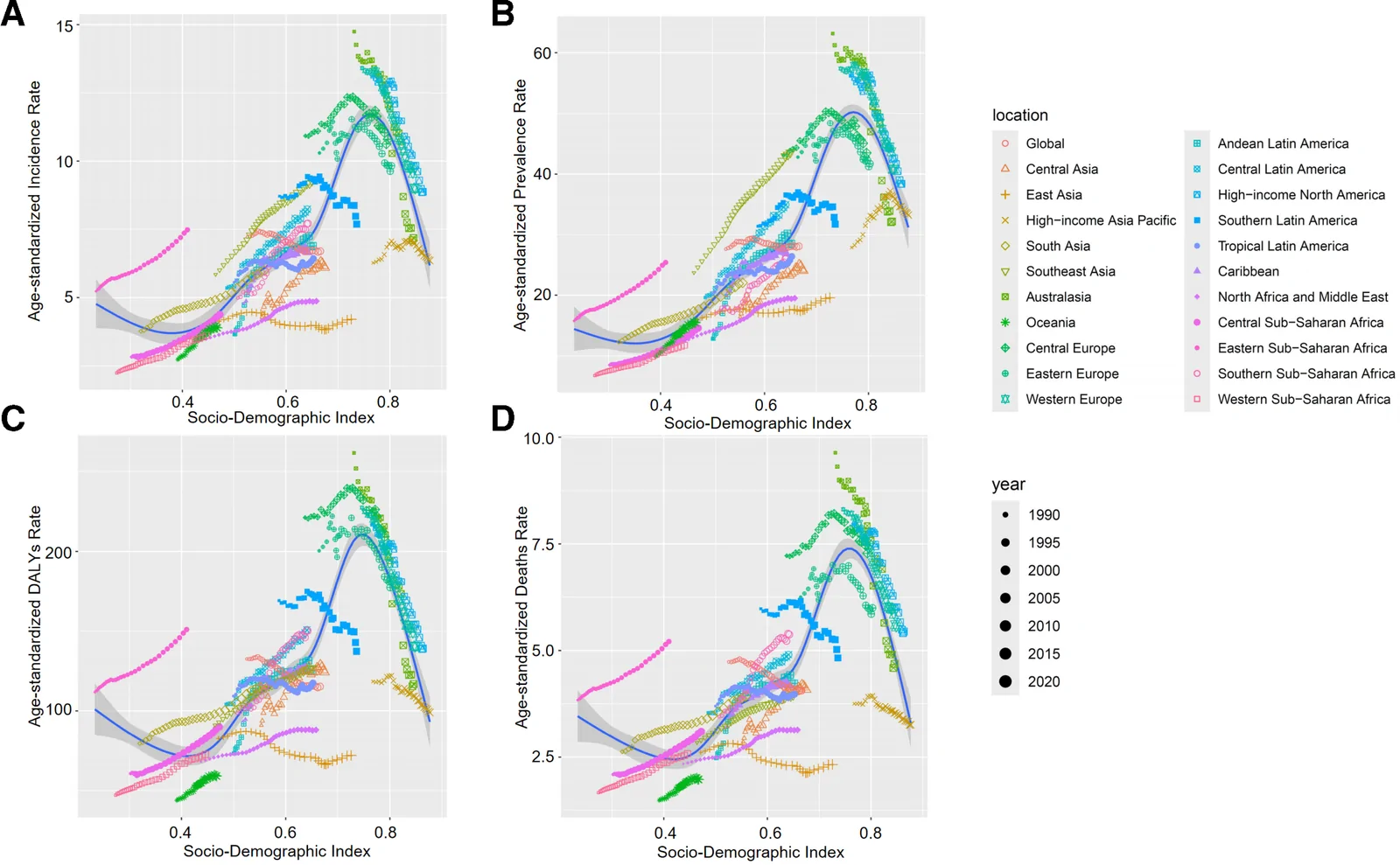
Trends in ovarian cancer burden and their relationship with SDI values across regions, 1990 to 2021. (A) Incidence rate, (B) Prevalence rate, (C) DALY rate, (D) Mortality rate (per 100,000 population). DALY = disability-adjusted life year, SDI = socio-demographic index.

### 3.4. Projected global trends to 2050

Forecasting with an autoregressive integrated moving average (ARIMA) model suggests that global age-standardized rates will remain broadly stable over the next 3 decades, although the absolute number of cases will continue to climb due to population aging and growth. In high-SDI regions, incidence and mortality are expected to maintain their downward trajectories. However, low- and low-middle-SDI regions are projected to experience sustained increases in incidence, prevalence, DALYs, and deaths through 2050. Without major improvements in prevention, screening, and treatment access, the overall burden of ovarian cancer is likely to continue shifting toward less developed areas, widening the global inequality gap in women’s cancer outcomes (Fig. 3).

**Figure 3.**
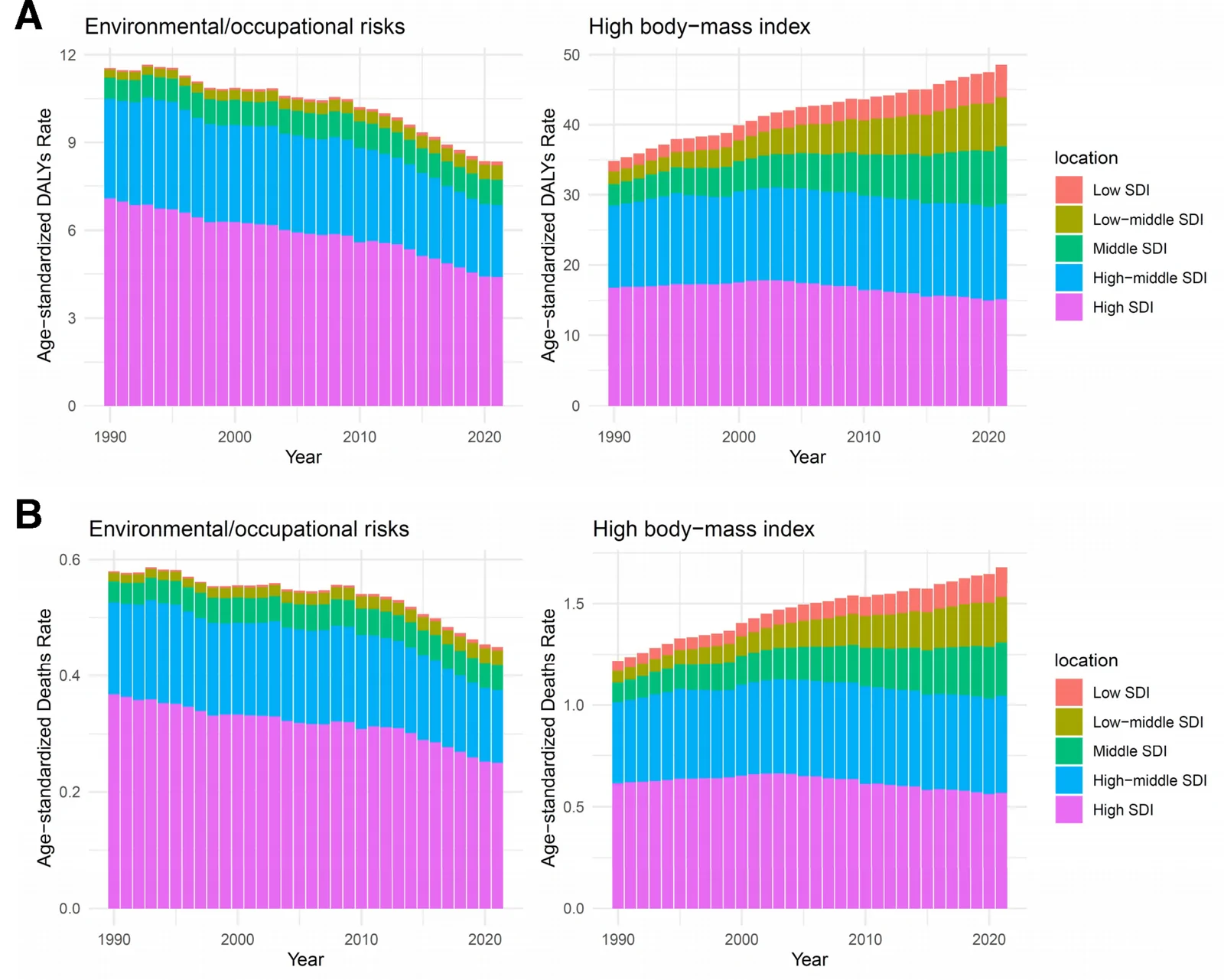
Trends and projections of age-standardized ovarian cancer burden across SDI regions, 1990 to 2050. (A) Incidence rate, (B) Prevalence rate, (C) DALY rate, (D) Mortality rate (per 100,000 population). DALY = disability-adjusted life year, SDI = socio-demographic index.

## 4. Discussion

This comprehensive analysis of the global, regional, and national burden of ovarian cancer from 1990 to 2021 reveals critical insights into disparities in incidence, prevalence, mortality, and DALYs. It underscores the multifaceted impact of sociodemographic factors, healthcare infrastructure, and public health policies on disease outcomes, providing a foundation for targeted interventions and future strategies.

A recent study by Xie et al also used GBD 2021 data to assess the burden of ovarian cancer and project trends to 2050.^[10]^ Several core estimates in our study were consistent with their findings, including the global ASIR, age-standardized prevalence rate , ASMR, and ASDR in 2021, suggesting that both analyses describe a highly comparable epidemiological pattern. However, our study focuses more specifically on the descriptive and policy-oriented interpretation of SDI gradients, age-specific burden, and regional disparities, with particular attention to the potential shift of future ovarian cancer burden toward less developed regions. Thus, this study does not merely duplicate previous work but provides an independent validation of the GBD 2021 estimates and further emphasizes their implications for health-resource allocation and public health planning.

### 4.1. Global disparities in ovarian cancer burden

The study identifies pronounced disparities in ovarian cancer burden across regions and SDI levels, with high-SDI regions exhibiting declining age-standardized rates for incidence, prevalence, DALYs, and mortality. This trend reflects the effectiveness of preventive measures, advancements in early detection, and accessibility to high-quality treatment in these regions. In contrast, low- and middle-SDI regions experience rising burdens, attributed to limited healthcare resources, inadequate screening programs, and delayed diagnoses.^[5]^

The increasing age-standardized incidence and mortality rates in low-middle SDI regions, coupled with the sharp rise in DALYs, highlight the urgent need for improved healthcare infrastructure and resource allocation. Low SDI regions, despite their lower absolute rates, face challenges in disease management and outcomes, further emphasizing global inequities.^[5,11]^

### 4.2. Age-specific burden and trends

Age-related trends in ovarian cancer burden align with known epidemiological patterns, where incidence and mortality rates rise with age. The observed peak in prevalence at 60 to 64 years and DALYs at 70 to 74 years reflect the late-stage diagnosis and high morbidity associated with the disease. These findings emphasize the critical need for targeted interventions in middle-aged and older populations, particularly in underserved regions where late-stage diagnoses are more prevalent.

### 4.3. Impact of sociodemographic index (SDI)

The association between ovarian cancer burden and SDI underscores the complex interplay between socioeconomic factors and health outcomes. Regions with moderate SDI values (0.71–0.82) bear the highest burden, likely reflecting a transitional phase where increasing life expectancy and changes in reproductive health patterns amplify disease risk. Conversely, regions with very low or very high SDI values show contrasting trends, highlighting the role of healthcare access and preventive measures.

Notably, the declining burden in high-SDI regions demonstrates the potential impact of comprehensive public health strategies, including widespread use of oral contraceptives, risk-reducing surgeries for high-risk individuals, and advancements in targeted therapies. These findings reinforce the importance of adapting similar strategies to resource-limited settings.^[12]^

### 4.4. Future projections and public health implications

Forecasts using ARIMA models indicate a continued rise in the ovarian cancer burden in low- and middle-SDI regions over the next 3 decades. This projected trend aligns with historical patterns, driven by aging populations, urbanization, and persistent gaps in healthcare access. In contrast, high-SDI regions are expected to sustain their declining trends, further widening the global disparity. To address these challenges, the study highlights several key recommendations^[13]^:

1. **Enhanced Screening and Early Detection:** Implementation of cost-effective, accessible screening programs tailored to regional needs is critical for reducing late-stage diagnoses.^[14]^
2. **Equitable Access to Treatment:** Expanding access to advanced surgical techniques, chemotherapies, and targeted treatments can significantly improve outcomes in underserved regions.
3. **Public Health Education:** Increasing awareness of ovarian cancer risk factors, symptoms, and preventive measures is essential for fostering early health-seeking behaviors.
4. **Investment in Research:** Further exploration of region-specific genetic, environmental, and lifestyle risk factors can inform more effective prevention strategies.

### 4.5. Limitations and future directions

While this study provides valuable insights, it is important to acknowledge limitations. Although the GBD 2021 framework applies multiple adjustment techniques and its reliability has been validated by previous studies, the estimates are derived from modeled data rather than direct measurements. Therefore, some degree of bias and uncertainty may still be inevitable. Variations in data quality and reporting accuracy across regions may influence the reliability of findings. Moreover, the study’s reliance on modeling for future projections introduces uncertainties, particularly in the context of evolving healthcare systems and policies.

Future research should focus on addressing these gaps by integrating primary data collection with robust epidemiological analyses. Additionally, evaluating the cost-effectiveness of interventions in low-resource settings will be crucial for guiding policy decisions.

## 5. Conclusion

This study underscores the urgent need for global and regional efforts to reduce the ovarian cancer burden, particularly in low- and middle-SDI regions where the disease continues to rise. By addressing disparities through targeted interventions, improved healthcare access, and sustained public health initiatives, policymakers and healthcare providers can make significant strides toward reducing the global impact of ovarian cancer.

## Acknowledgements

We thank all the individuals who contributed to the Global Burden of Disease Study 2021 for their extensive support in finding, cataloguing, and analyzing data and facilitating communication between and among team members.

## Author contributions

**Formal analysis:** Yajing Zhang, Xike Zhang.

**Writing – original draft:** Chaokang Huang, Xin Zheng.

**Writing – review & editing:** Chaokang Huang, Jian Zhang.
